# First prospective comparison of genotypic vs phenotypic tropism assays in predicting virologic responses to Maraviroc (MVC) in a phase 3 study: MODERN

**DOI:** 10.7448/IAS.17.4.19519

**Published:** 2014-11-02

**Authors:** Jayvant Heera, Srinivas Valluri, Charles Craig, Annie Fang, Neal Thomas, Ralph Dan Meyer, James Demarest

**Affiliations:** 1 Clinical Development, Pfizer Inc, Groton, USA; 2 Statistics, Pfizer Inc, New York, USA; 3 Virology, Pfizer Inc, Sandwich, UK; 4 Clinical Development, Pfizer Inc, New York, USA; 5 Statistics, Groton, Pfizer Inc, USA; 6 Virology, ViiV Healthcare, Research Triangle Park, USA

## Abstract

**Introduction:**

MODERN (A4001095) was the first prospective phase 3 study comparing genotype vs phenotype (Trofile™) tropism assessments.

**Materials and Methods:**

Treatment-naïve adults with HIV-1 RNA >1000 copies/mL were randomized 1:1 at screening to either genotype or Trofile for tropism assessment. Genotype was determined using the geno2pheno algorithm to assess triplicate HIV-1 gp120 V3 loop sequences (plasma); false-positive rate=10%. R5-virus-infected subjects were then randomized 1:1 to receive Maraviroc (MVC) 150 mg QD or Truvada 200/300 mg QD each with DRV/r 800/100 mg QD. Tropism of screening samples from enrolled subjects was also retrospectively determined using the alternate testing method. Positive predictive values (PPV) were estimated by%R5 subjects with Week 48 HIV-1 RNA < 50 c/mL. PPV for each assay was estimated using the response rate among those randomized to that assay and using model-based response estimates in those with R5 by that assay (at screening or retest).

**Results:**

The observed response rate was 146/181 (80.7%) for genotype vs 160/215 (74.4%) for Trofile (stratification adjusted difference=6.9%, 95% CI 1.3% to 15%). The model-based estimates of PPV (±SE) were 79.1% (±2.42) and 76.3% (±2.38), respectively (difference = 2.8%, 95% CI −2.1% to 7.2%). There was no difference in response rate between assays in the Truvada arm (observed difference=− 0.1%, 95% CI −6.8% to 6.6%). Most enrolled subjects had R5 results at screening using both assays (285/396 (72%)), and of these subjects, 79.3% (226/285) had HIV-1 RNA <50 c/mL at week 48 (Table 1). The few subjects classified as non-R5 by the alternate assay had similar virologic responses to the concordant R5 group.

**Conclusion:**

There was a higher MVC response rate and model-based positive predictive value with genotype compared to Trofile, but this difference did not reach statistical significance. The majority of subjects had concordant R5 tropism results. Either phenotype or genotype can effectively predict MVC response.

**Table 1:** MVC week 48 response (HIV-1 RNA<50c/mL), *n*/*N* (%)

MVC week 48 response (HIV-1 RNA<50c/mL), *n*/*N* (%)	Trofile R5	Trofile non-R5	Trofile non-reportable	Total
Genotype R5	226/285 (79.3)	11/14 (78.6)	14/19 (73.7)	251/318 (78.9)
Genotype non-R5	21/26 (80.8)	0	0	21/26 (80.8)
Genotype non-reportable	34/52 (65.4)	0	0	34/52 (65.4)
Total	281/363 (77.4)	11/14 (78.6)	14/19 (73.7)	306/396 (77.3)

